# The New Tri-State Directory

**Published:** 1912-01

**Authors:** 


### The New Tri-State Directory

One of the obligations which was assumed by the Medical Society of the State of New York in securing the fusion with it of the seceding Association, was the continuance of the medical directory instituted by the latter organization. At the time of the reunion of the profession of the state, the writer remarked that about all that the Society retained was its title and its hundred years of existence—although it should be stated that this remark was made with no bitterness of feeling and with no implication of regret. Many of the members of the Society have undoubtedly felt that the publication of the directory involved an expense disproportionate to the benefit conferred. Without wishing in any way to make an issue of this point, it may be pointed out that one of the most necessary details in fostering a spirit of professional harmony and fraternalism, is a ready means of keeping in touch with the profession. To have this means in the hands of every physician who takes enough interest in his profession to affiliate himself with its general organizations, tends to preserve ties of friendship, to facilitate correspondence with regard to cases, discussion of published articles etc., and to render it easy to get together, literally or by correspondence, in the many instances in which it is desirable to secure joint action on important matters of professional and general interest.

For the average physician, and for the average emergency requiring coöperation, this directory answers the purpose. Only occasionally, is it necessary to refer to the larger, more cumbrous and much more expensive directories of national scope.

The present directory occupies about 800 pages net, due to slight condensation of matter, in spite of the increase from 17,416 to 17,624 names for the three states, New York, New Jersey and Connecticut.

We have previously referred to the economic side of statistics of general and of medical population. The profession of this state has increased by 167 from 1910 to 1911—a trifle over 1 1-5 per cent. The total population is increasing at the rate of nearly 2 per cent. a year—over 25 per cent. from 1900 to 1910, but we must allow for "compounding" as in calculating interest. Last year, there was one physician to every 676 of population. This year, if we estimate an even 2 per cent. of increase of population, the ratio is 681. Outside of greater New York, there were 6,392 physicians in 1911, 6,305 in 1910, giving approximately the same ratios.

It is interesting to compare these statistics with others. Pope listed 13,344 physicians for New York State in 1910—130 less than the tri-state directory—the highest number being for 1906—14,238—while the total of *registered* physicians for 1911 given by the tri-state directory (13,461) is just three more than the listing of Pope for 1904.

All these statistics warrant the optimistic belief that educational requirements and a general disposition to recognize and combat the excess of supply over demand, are really accomplishing results. But we are still far from having realized a condition of equilibrium and the favorable tendency should only stimulate more energetic measures toward securing the economic welfare of the profession.

We would urge the members of the profession to use the directory as a means of keeping in touch with old friends who have removed, and in the other ways suggested. It is important to have the directory as accurate as possible and the BUFFALO MEDICAL JOURNAL will gladly act as a bureau for recording deaths, removals, corrections, additions, etc., for western New York and will transmit the results to the directory committee for the next edition.

